# Trafficking of a nitrogenase FeMo-cofactor assembly intermediate

**DOI:** 10.1038/s41589-026-02179-0

**Published:** 2026-03-23

**Authors:** Florian F. Schneider, Julia S. Martin del Campo, Lin Zhang, Dennis R. Dean, Oliver Einsle

**Affiliations:** 1 Institute of Biochemistry, Albert-Ludwigs-Universität Freiburg, Freiburg, Germany; 2 Department of Biochemistry, Virginia Tech, Blacksburg, VA USA; 3 Faculty of Synthetic Biology, Shenzhen University of Advanced Technology, Shenzhen, China

**Keywords:** Metalloproteins, Structural biology

## Abstract

The maturation of the unique FeMo-cofactor of molybdenum nitrogenase is a multistep process requiring the sequential action of a series of maturase complexes. As a final step, the NifEN complex forms FeMo-cofactor from the precursor NifB-co, also called L-cluster, through replacement of an apical iron ion by molybdenum and the attachment of an organic homocitrate ligand. NifB-co is delivered by a small cofactor chaperone, NifX, and initially bound near the surface of the maturase NifEN. Here, we report high-resolution cryo-electron microscopy structures of NifEN in complex with NifX, showing NifB-co binding to NifEN in full detail, capturing both interacting partners in the act of cluster transfer. In a dynamic transfer complex, the large metal cluster is coordinated by single residues from both NifEN and NifX. In silico studies concur with these structures but suggest a third, internal conversion site where cluster maturation likely takes place.

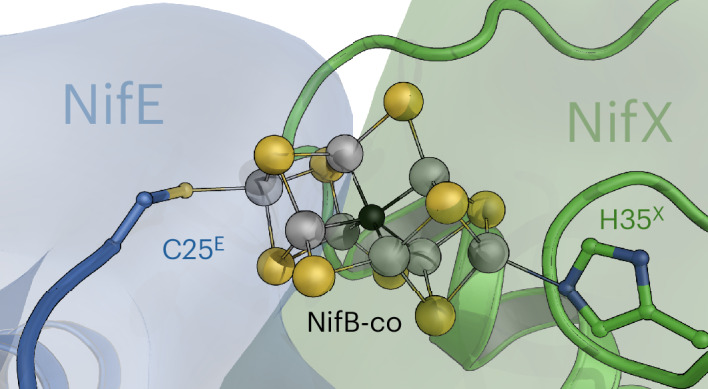

## Abstract (web summary)

The maturation of the unique FeMo-cofactor of molybdenum nitrogenase is a multistep process requiring the sequential action of a series of maturase complexes. Here, the authors report on how cryo-electron microscopy structures show NifB-co transfers from NifX to NifEN’s internal site, where NifB-co is converted into FeMo-co for insertion into Mo-nitrogenase.

## Main

Biological nitrogen fixation is the essential and unique microbial process that converts atmospheric nitrogen (N_2_) into ammonium (NH_4_^+^), the most reduced modification that is bioavailable for incorporation into organic molecules. Catalyzed by the nitrogenase enzyme complex, this process has a critical role in the global nitrogen cycle, contributing to the nitrogen needs of plants and, by extension, sustaining ecosystems and agricultural productivity^1^. Nitrogen fixation is carried out by a wide range of microorganisms, including free-living marine and soil bacteria, symbiotic nitrogen-fixing bacteria in root nodules and cyanobacteria. The importance of nitrogen fixation is underscored by its contribution to maintaining soil fertility and providing a renewable source of nitrogen in ecosystems where nitrogenous compounds are otherwise scarce. Given its biological importance, the molecular mechanisms underlying nitrogen fixation have remained a subject of intensive research, particularly regarding the assembly of the enzymatic machinery that facilitates the reduction of atmospheric N_2_ (refs. ^2–6^).

**Fig. 1:**
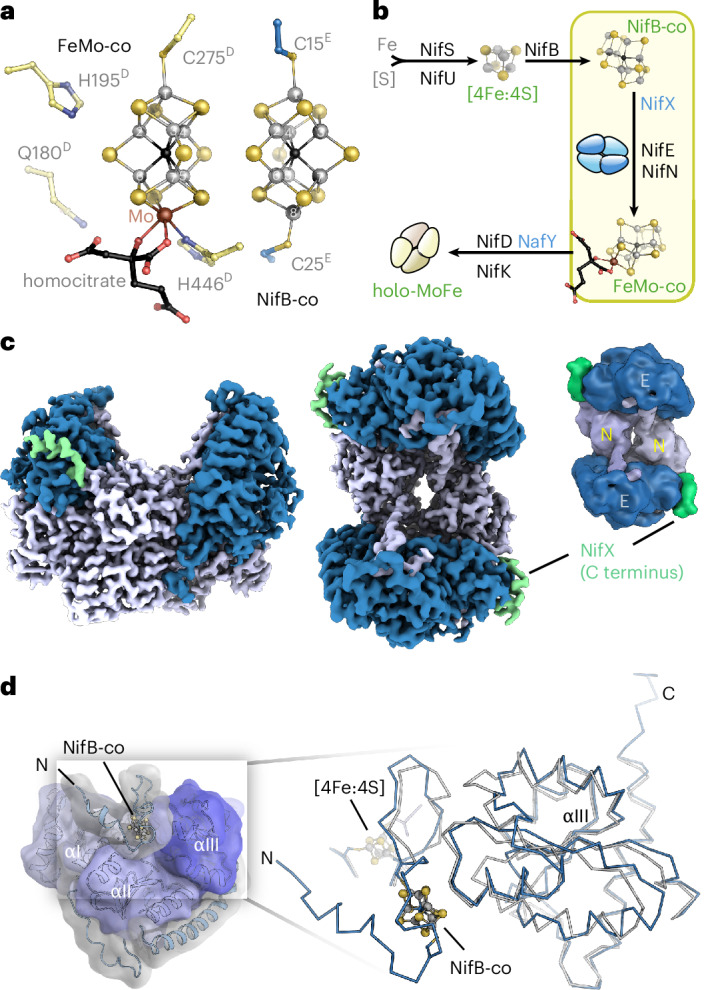
The NifEN complex in FeMo-cofactor maturation. **a**, Mo-nitrogenase cofactors. The active site FeMo-co is assembled along a complex pathway ex situ and only inserted into apo NifDK as the final step of assembly. The key intermediate of cofactor assembly is NifB-co (L-cluster), whose final modification steps take place at the NifEN complex. **b**, Overview of maturases and chaperones involved in FeMo-co biogenesis. The NifSU machinery produces [4Fe:4S] cubanes. The maturase NifB fuses two of them and inserts a carbide from SAM and a sulfide to yield the [8Fe:9S:C] NifB-co. NifX shuttles NifB-co to the NifEN complex, where Mo and homocitrate are inserted before the chaperone NafY transports and inserts the complete FeMo-co into apo NifDK. **c**, Cryo-EM maps for *A*. *vinelandii* NifEN at 2.14-Å resolution in front and top view. NifE, dark blue; NifN, light blue. The resolved N termini of two bound NifX proteins are shown in green. **d**, Three-domain architecture of NifE and overlay of the C_α_ backbone of the NifEN cryo-EM structure with the crystal structure (PDB 3PDI) for the third Rossmann fold domain of NifE (αIII) that is most relevant to NifB-co binding.

The most thoroughly studied isoform of nitrogenase depends on molybdenum as a heterometal in its active site cofactor for catalytic activity and is the focus of the present work^7^. Alternative but closely related isoforms substitute Mo by either vanadium or iron and show variations in their corresponding biogenesis pathway^3,4,8^. Molybdenum nitrogenase is a multisubunit enzyme complex composed of two key components: the Fe protein (or dinitrogenase reductase) and the MoFe protein (or dinitrogenase)^3^. The homodimeric Fe protein is responsible for delivering electrons to the MoFe protein^9^, where the reduction of substrates takes place^6,10^. The MoFe protein is a 230-kDa dimer of heterodimers consisting of two α-subunits and two β-subunits (NifD_2_K_2_) and houses the electron-transferring [8Fe:7S] P-cluster and the FeMo-cofactor (FeMo-co), a unique [Mo:7Fe:9S:C]:homocitrate cluster essential for dinitrogen reduction (Fig. 1a)^11,12^. The biogenesis of this FeMo-cofactor is a tightly regulated process, involving a series of biosynthetic steps that occur ex situ on scaffold proteins, connected by small carriers or chaperones that shuttle intermediates of the complex moiety (Fig. 1b). The assembly of the FeMo-cofactor begins with the formation of cubane-type [4Fe:4S] clusters through the interplay of the cysteine desulfurase NifS with the first assembly scaffold, the NifU protein^13^. Two such cubane units are transferred to the radical-SAM family enzyme NifB that inserts a carbide ion originating from *S*-adenosyl methionine and a further sulfide, rearranging two [4Fe:4S] clusters into a [8Fe:9S:C] precursor termed NifB-co or L-cluster (Fig. 1a)^14^. In the alternative Fe-nitrogenase, NifB-co is only further modified by the addition of a homocitrate ligand to its apical Fe8 ion. However, both the Mo-dependent and V-dependent isoforms fully replace Fe8 with a Mo or V ion, respectively, before or in concert with the bidentate ligation by homocitrate^3^.

For Mo-nitrogenase, substitution of an apical Fe in NifB-co and attachment of homocitrate occur on the NifE_2_N_2_ complex, a structural homolog of MoFe protein^15^. A similar complex, VnfEN, is present in the gene cluster encoding vanadium nitrogenase and is presumed to replace Fe for V instead^16^. A 2011 crystal structure of *Azotobacter* *vinelandii* NifEN revealed a similar architecture to MoFe protein, as well as the replacement of the nitrogenase P-cluster by a canonical [4Fe:4S] cluster, corresponding to the half of P-cluster that is bound to NifD, in the analogous position at the NifE–NifN interface^17^. The structure also showed a large electron density peak consistent with a moiety suggested to be NifB-co bound to the surface of NifE, close to its N terminus. However, the exact nature of this cluster and the mode of its ligation to the protein were not resolved. The authors hypothesized that Mo replacement of an apical Fe within NifB-co and attachment of homocitrate would occur at a position in the NifEN scaffold similar to the one occupied by FeMo-co in MoFe protein but the sequence of events and the structures of assembly intermediates remain to be clarified^17^. As an additional level of complexity, a gene located immediately downstream of *nifEN*, *nifX*, encodes a protein (NifX) having the capacity to bind both NifEN and NifB-co, which is proposed to shuttle NifB-co from its assembly site within NifB to NifEN^18^, although details of this process at the molecular level have remained unclear. A corresponding role has been suggested for NafY, which is proposed to shuttle completed FeMo-cofactor from the NifEN scaffold to apo NifD_2_K_2_ (ref. ^19^).

In this work, we set out to gain further insight into the functionality and interaction of NifEN. To this end we prepared the NifEN–NifX complex from *A*. *vinelandii*, determined structures using cryo-electron microscopy (cryo-EM) single-particle analysis and obtained two different structural states with respect to bound cofactor and the role of NifX. Our data show that NifX is tightly tethered to the NifEN complex through its C terminus but retains sufficient flexibility to accept NifB-co from NifX and shuttle it to a receiving site near the surface of NifE that, however, likely is not the site of its conversion into FeMo-co.

## Results

### Structure of cofactor-loaded NifEN

**Fig. 2:**
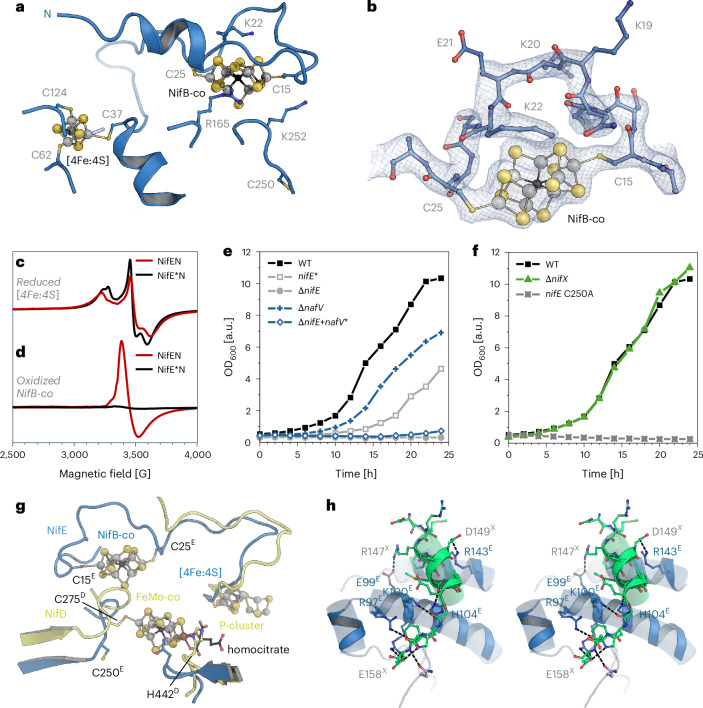
NifB-co binding to NifEN. **a**, Residues C15^E^ and C25^E^ act as apical ligands to bound NifB-co, structuring the otherwise disordered N terminus of NifE. **b**, Coulomb map for the N terminus of NifE with bound NifB-co. **c**, X-band EPR spectra of reduced NifEN and NifE*N show the presence of the [4Fe:4S] cluster in both samples. **d**, In contrast, only NifEN binds NifB-co, as evidenced by the absence of the EPR signature of the cluster in NifE*N. **e**, Diazotrophic growth of *A*. *vinelandii* and variants. The NifE* variant shows reduced growth, while Δ*nifE* does not growth diazotrophically. Deletion of NafV, the electron donor for NifB, substantially slows diazotrophic growth and renders the N terminus of NifE essential. **f**, Deleting NifX does not affect diazotrophic growth, likely because of the compensatory effect of other NifX-like proteins. In contrast, residue C250^E^ is essential. **g**, Comparison of cofactor binding in NifEN (blue) and the mature MoFe protein, NifDK (yellow). In NifEN, NifB-co is bound by the N terminus of NifE, residing on the surface of the maturase. In NifDK, FeMo-co is inserted deeply into the protein and cradled between the three Rossmann fold domains of NifD. The corresponding cofactor cavity is present in NifEN, including a putative cysteine ligand to Fe1 (C250^E^) but remains unoccupied in the structure obtained immediately following cluster transfer. **h**, Wall-eyed stereo representation of the tight interaction of the C terminus of NifX with NifE. Several salt bridges provide notable binding strength.

We refined three-dimensional (3D) classes containing cofactor-loaded NifEN, as well as NifEN with a single bound NifX that—while intrinsically flexible—was modeled and reveals the mode of NifB-co binding to this chaperone before its transfer to the maturase NifEN. The structure of NifEN at 2.14-Å resolution confirmed the earlier crystal structure analysis^17^, with an overall root-mean-squared deviation (r.m.s.d.) of 2.4 Å for all atoms (Fig. 1c). The NifE_2_N_2_ heterotetramer is built around a tight dimer of NifN, stabilized by two Mg^2+^ ions at the interface of the NifN dimer, and each NifN is in contact with one of the NifE subunits. This architecture is similar to the three dinitrogenase isoenzymes but the present cryo-EM structure resolves further details that were not seen in the earlier X-ray diffraction structure. In the reported crystal structure, the C terminus of NifN was only resolved up to residue H435^N^, the new data now extend to residue P449, just eight residues from the end of the protein chain that remained flexible (Fig. 1c and Extended Data Fig. 2a,b). This part of NifN bridges a gap without contact to either subunit, before tightly interacting with NifE (Extended Data Fig. 2c). The absence of the NifN C terminus in the crystal structure might indicate a dynamic interaction during the complex cofactor maturation process. Further differences between the two NifEN structures are largely limited to the environment of bound NifB-co at the third of three Rossmann fold domains in NifE, αIII, a region of increased flexibility (Fig. 1d). The cryo-EM structure fully resolves the N-terminal segment of NifE that binds and shields the bound NifB-co, including M1, with a short stretch of α-helix from residues K4^E^ to L10^E^ (Fig. 2a), and confirms cysteine residue C25^E^ as a ligand to an apical Fe ion (Fe8). Toward the N terminus, the NifE chain wraps around the cluster and provides cysteine C15^E^ as a ligand to Fe1 at the other apex of the moiety (Fig. 2b). Although it was bound at the surface of NifEN, the cluster was effectively shielded from the environment by the protein chain. Bound NifB-co can be unambiguously identified as a complete [8Fe:9S:C] moiety, including three µ_2_-bridging belt sulfides and a central carbide (Fig. 2b and Extended Data Fig. 3). In a sharpened map, the density for the central carbide disappeared, which, at the given resolution of 2.14 Å, is because of the same scattering artifacts that obscured the central light atom in the initial crystal structures and originate from resolution-dependent Fourier series termination errors^12^. The in vitro and in vivo contribution of this NifB-co-docking site to FeMo-cofactor formation was evaluated by constructing a strain that produces a truncated NifE having residues 4–25 removed, including the NifB-co binding residues C15^E^ and C25^E^. Hereafter, the form of NifEN without the N-terminal NifB-co-binding loop is designated NifE*N. A comparison of the electron paramagnetic resonance EPR spectra of isolated NifEN and NifE*N revealed approximately the same [4Fe:4S] cluster occupancy for NifE*N as for wild-type NifEN, albeit exhibiting modest changes in *G* values and line-shape (Fig. 2c). In contrast, the EPR signature of NifB-co, evident within wild-type NifEN, is absent in the NifE*N species (Fig. 2d). Consistent with these observations, the isolated wild-type NifEN tetramer contained 19 ± 1 Fe of the potential 24 Fe atoms expected for full occupancy of the [4Fe:4S] cluster and NifB-co, whereas NifE*N contained only 8 ± 1 Fe atoms, consistent with full occupancy of the [4Fe:4S] sites and no binding of NifB-co. It was further shown that the NifB-co-loaded form of isolated wild-type NifEN but not the altered NifE*N could support in vitro apo NifD_2_K_2_ maturation, yielding an acetylene-reducing activity of the resulting reconstituted Mo-nitrogenase of 352 ± 11 nmol ethylene min^−1^ mg^−1^.

The NifE*N-producing strain retained a capacity for diazotrophic growth when shifted to a condition that demands N_2_ fixation (Fig. 2e, ο) but only after an extended lag time when compared to the wild-type strain (Fig. 2e, ν). The diazotrophic growth capacity of the NifE*N strain cannot be attributed to alternative nitrogenases, because a strain harboring a large deletion within *nifE* had no capacity for diazotrophic growth when cultured under the same conditions (Fig. 2e, λ). These results indicate that the NifB-co binding site within the N-terminal domain of NifE, which is conserved in all available NifE sequences, has a critical role in supporting FeMo-cofactor formation during transition to diazotrophy, when the pool of available NifB-co is relatively low. This possibility was tested by construction of an *A. vinelandii* strain that combined a mutation within NafV (FdxN) with the truncated NifE* (Fig. 2e, +). NafV is a ferredoxin required for effective formation of NifB-co and its inactivation results in a fivefold depletion in NifB-co accumulation^18^. Expression of NifE*N in combination with inactivation of NafV eliminated the capacity for diazotrophic growth (Fig. 2e, υ), confirming an essential role for the N-terminal NifB-co-binding site within NifE when the availability of NifB-co is limited. Interestingly, we find that NifX is not required to support diazotrophic growth and is, therefore, not essential for FeMo-cofactor formation on the NifEN scaffold (Fig. 2f)^20^. This result is not surprising given that a variety of other proteins in *A*. *vinelandii* have primary sequence and predicted structural similarity to NifX (Extended Data Fig. 4), including the analogous NifB-co binding H35 residue present in NifX, are also produced under diazotrophic conditions^21^. The most striking of these is the NifX-like domain located at the C terminus of NifB, the protein scaffold upon which NifB-co is formed before its delivery to the NifEN complex (Extended Data Fig. 4b)^22^. It also includes NafY, a FeMo-co chaperone that delivers FeMo-co after the completion of its assembly on NifEN to apo NifDK (Extended Data Fig. 4c)^23,24^, and the VnfX protein that shuttles NifB-co to the VnfEN complex during the maturation of V-nitrogenase (Extended Data Fig. 4d)^25^. Other members of the family, including NifY^26^ and NafX^21^, lack the cofactor-binding histidine but retain the overall fold, which might act as placeholders or antagonists in the maturation process (Extended Data Fig. 4e,f).

The structural similarity between NifEN and MoFe protein extends further than the overall shape and architecture of both proteins. In mature MoFe protein, the FeMo-cofactor is bound far from the protein surface, in a large internal cavity surrounded by all three Rossmann fold domains of NifD. In NifEN, this unoccupied cavity is retained and is present as a cavernous space within the scaffold protein, situated directly below the position of NifB-co bound to the protein surface (Fig. 2g). One of the ligands to FeMo-cofactor in MoFe protein, the apical C275^D^, is conserved as C250^E^ in NifEN (Fig. 2a,g). However, all other residues known to be indispensable for nitrogenase activity are not conserved. H442^D^, a ligand to the molybdenum ion, is replaced by Q419^E^, as are the essential active site residues V70^D^ (I44^E^), Q191^D^ (K160^E^) and H195^D^ (N164^E^). Notably, substitution of NifE C250^E^ by A250^E^ inactivates the capacity to form FeMo-cofactor (Fig. 2f), supporting the original suggestion that this residue has an important role in FeMo-cofactor formation within the NifEN scaffold^15^. The linear coordination of NifB-co by two cysteine residues emphasizes that the insertion of molybdenum and addition of homocitrate have not yet taken place. We, therefore, suggest that NifB-co binding at the N terminus of NifE represents an early event in the catalytic sequence of the maturase NifEN.

### The NifENX complex and cofactor transfer

**Fig. 3:**
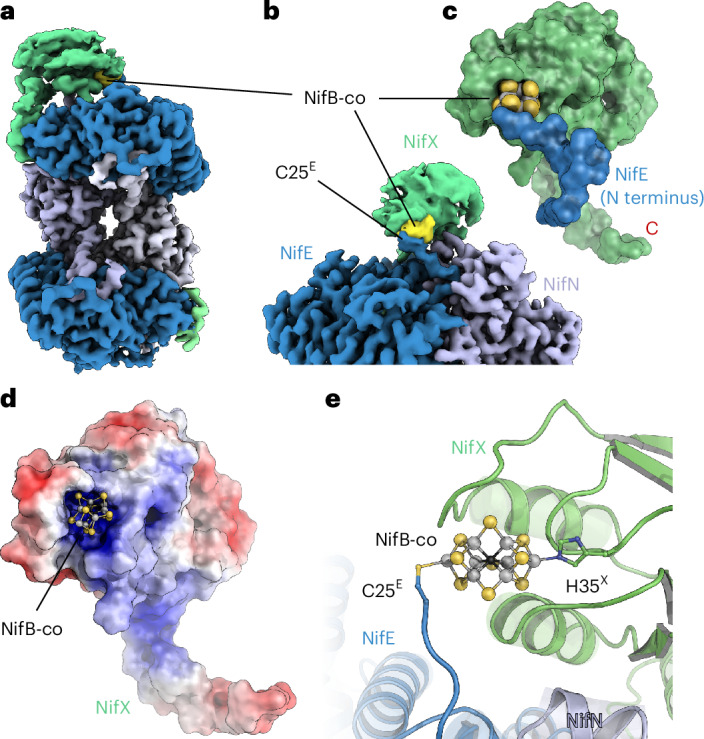
NifEN interaction with NifX and transfer of NifB-co. **a**, Map of the 3D class with NifX present. The map indicates a degree of flexibility of NifX, whose globular domain is only resolved on one side of the *C*_2_-symmetric NifEN. **b**, Density for bound NifB-co on NifX, contacted directly by NifE. **c**, Structural model of the handover complex for NifB-co, with NifX as ligand to one apical Fe of the cofactor precursor and the N terminus of NifE to the other. **d**, Electrostatic surface potential for NifX, contoured from −5 *k*_B_*T* (red) to +5 *k*_B_*T* (blue). NifB-co is bound to a distinctly positively charged binding pocket. **e**, Coordination of NifB-co in the handover complex. The N terminus of NifE is disordered up to residue C25, which serves as an apical cluster ligand. In NifX, H35 coordinates the other apex of NifB-co.

**Extended Data Fig. 1:**
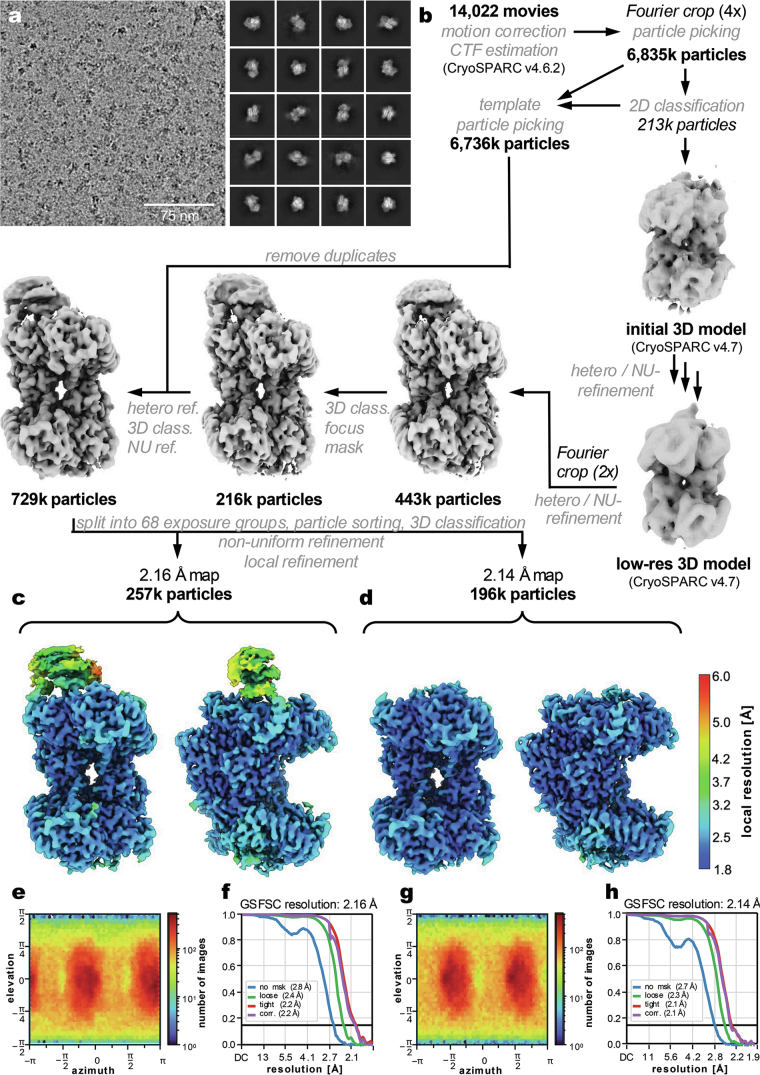
Workflow for the cryo-EM structure determination of NifEN and NifENX. **a**, Representative micrograph and 2D class averages from 14,022 recorded movies. **b**, 6,835,000 particles were picked for 2D classification and initial 3D reconstruction and subsequently split into two 3D classes representing: **c**, a NifE_2_N_2_X from 257k particles and **d**, a NifE_2_N_2_ structure from 262k particles. The obtained local resolution was similar for the NifE_2_N_2_ core but increased for the resolved NifX monomer. **e**, Angular distribution of particles used for the 3D reconstruction, and **f**, FSC curves (threshold 0.143) for NifE_2_N_2_X. **g**, Angular distribution of particles used for the 3D reconstruction and **h**, FSC curves (threshold 0.143) for NifE_2_N_2_.

**Extended Data Fig. 2:**
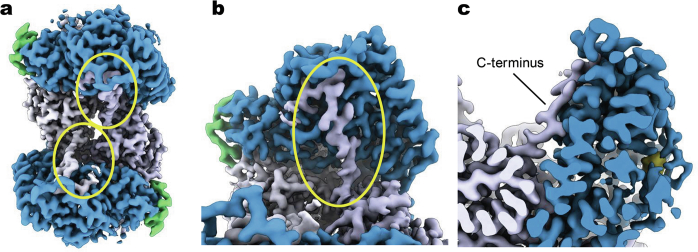
C-terminus of NifN. **a**, In the cryo-EM maps of NifEN and NifENX, the C-terminus of NifN was ordered, in contrast to the earlier XRD analysis. **b**, In each half on NifEN, the C-terminus of NifN extended straight towards an adjacent NifN subunit. **c**, Side view of NifEN, sliced for clarity. The C-terminus of NifN bridges a gap to contact NifE, providing additional stability to the heterotetrameric maturase.

**Extended Data Fig. 3:**
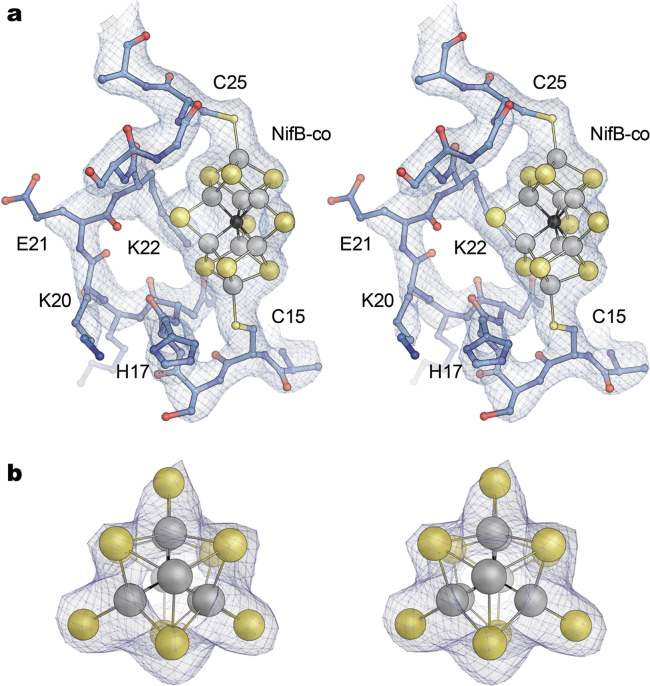
Representative 3D maps for NifB-co in wall-eyed stereo. **a**, The cofactor-binding loop on NifE with bound NifB-co. **b**, A view along the threefold axis of the cofactor. All µ_2_-bridging sulfides are present.

**Extended Data Fig. 4:**
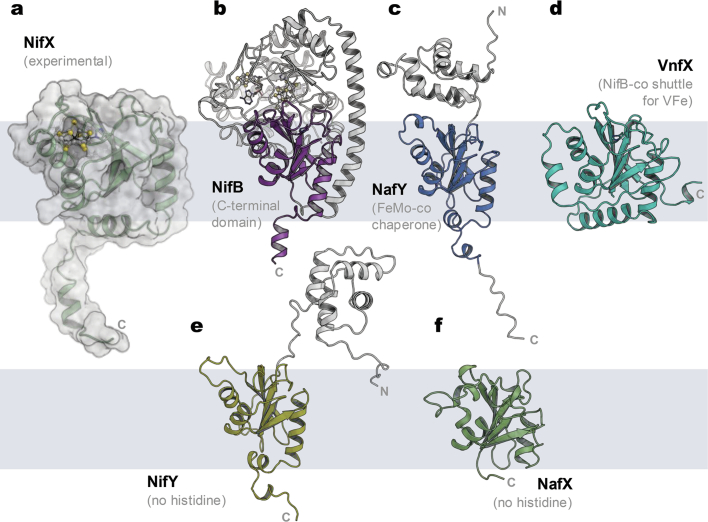
NifX-like proteins in *A. vinelandii.* **a**, Experimental structure of NifX from the present work. All following models were generated with AlphaFold3. **b**, The cofactor maturase NifB, with a C-terminal NifX-like domain (purple). **c**, NafY, the cofactor chaperone that shuttles mature FeMo-co from NifEN to apo-NifDK. The C-terminal NifX-like domain (blue) contains a cofactor-binding histidine. **d**, VnfX is a NifX-like protein in the *vnf* gene cluster for V-nitrogenase, supposed to interact with the VnfEN complex, analogous to NifX (teal). **e**, The two-domain NifY protein has a C-terminal NifX-like domain but lacks the cofactor-binding histidine. **f**, NafX is a further NifX-like protein of unknown function, lacking the histidine required for cofactor binding.

**Extended Data Fig. 5:**
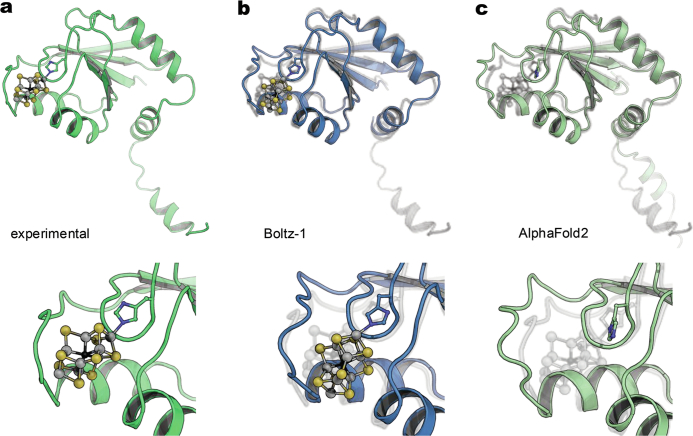
Experimental structure of NifX in the NifENX complex and *in-silico* models. Overall structures are shown above, an enlarged view of the cluster environment is below. The experimental structure is shown as a greyed-out overlay with the two predicted structures. **a**, Refined model based on experimental data. A starting model was generated using AlphaFold3 (c). **b**, The Boltz-1 server allows for the free definition and inclusion of ligands into structure predictions. It correctly identified H35^X^ as a ligand to the cluster and placed the moiety in a similar orientation to the one observed in the NifENX complex. **c**, The largest difference between the AlphaFold-generated starting model and the experimental structure is seen in the elongated loop adjacent to the cluster binding site. This may well be due to the absence of the cluster, as it is less pronounced in the cluster-containing model generated by Boltz-1 (b).

**Extended Data Fig. 6:**
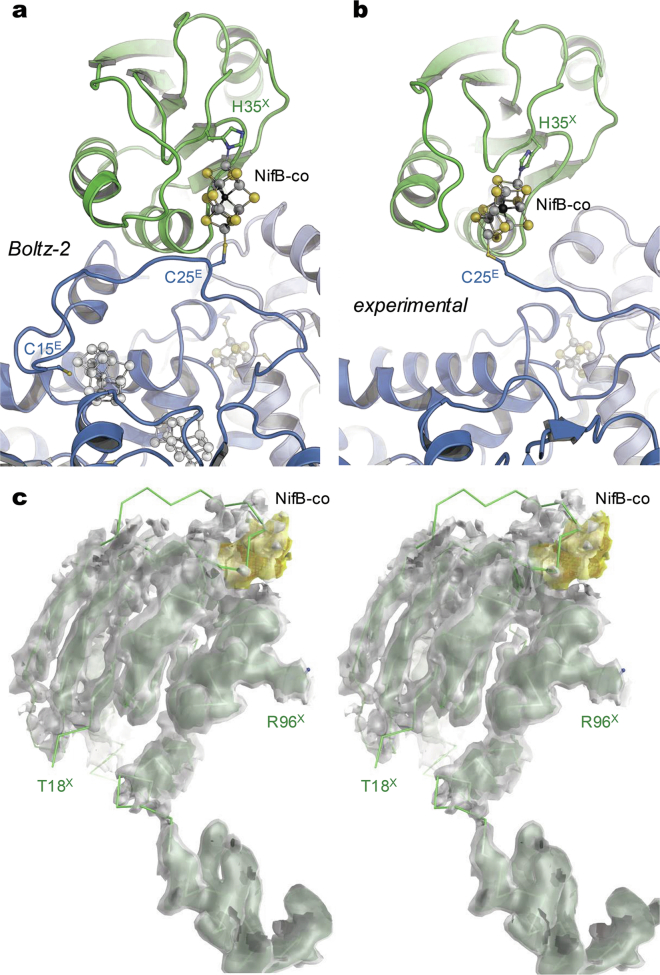
*In-silico* prediction vs. experimental structure of the NifX-NifEN handover complex. **a**, When asked to include three copies of NifB-co, Boltz-2 in most cases had the clusters occupy the sites on NifX and the receiving and conversion site on NifE. Rarely, however, one cofactor precursor was placed in the bridging position observed in the experimental structure. **b**, Experimental structure of the NifENX handover complex. **c**, Stereo depiction of the experimental density map for the NifX subunit with the structural model shown as a C_α_ trace. Maps are contoured at 2.5σ (white) and 3.5σ (green). Bound NifB-co is shown in yellow. The N-terminus at T18^X^ and the prominent R96^X^ are shown as sticks.

For structure determination, NifEN was isolated together with the NifB-co chaperone NifX. The 3D classes containing surface-bound NifB-co did not show density for NifX, with the notable exception of the very C terminus of the chaperone. The terminal 15 residues, D144^X^ to E158^X^, were well defined and specifically bound to a patch with positive electrostatic surface potential on the αI domain of NifE. Strong, ionic interactions were formed with R97^E^, E99^E^, K100^E^, R101^E^ and H104^E^, with an additional, single hydrogen-bonding interaction with N8 of NifN (Fig. 2h). This implies that the N-terminal portion of NifX was obviously present but disordered. This situation was different in a second 3D class from the same dataset, where a single NifX was defined. While still flexible, an in silico NifX model could be straightforwardly fit into this map (Fig. 3a). In the 2.16-Å resolution structure, the N terminus of NifX was disordered up to residue T18^X^, in line with low predicted local distance difference test scores obtained in AlphaFold3 (Fig. 3b,c and Extended Data Fig. 5c). NifX folds into a globular domain with β_2_(βα)_3_ topology and an overall rigid folding core. Following the terminal helix of the globular domain terminating at residue N126^X^, two additional α-helices, α4 and α5, form the C terminus of NifX, connected by flexible loops, with α5 binding tightly to NifE (Fig. 2h and Extended Data Fig. 5b). While this binding established a strong link between both proteins, the NifX domain itself remained flexible with respect to NifEN, which was apparent in a lower local resolution of the map in the range of 3–5 Å (Extended Data Fig. 1c). Nevertheless, the overall structure of NifX could be well resolved and the density map showed a substantial peak consistent with the presence of a bound NifB-co at NifX rather than at the surface of NifEN (Fig. 3b,c and Extended Data Fig. 6). The total charge of NifB-co bound to NifEN is not well established; however, with nine formal sulfides (S^2−^) and the central carbide (C^4−^), a nominal charge of −2 would remain even if four of the eight iron ions were in a nominally oxidized Fe^3+^ state. It is, therefore, not surprising that the environment of the bound cofactor on NifE is rich in positively charged residues (Fig. 3d), with K22^E^ and R165^E^ in the immediate vicinity of the site (Fig. 2a).

The N terminus of NifE was disordered up to residue C25^E^, which was close to the density maximum for the cluster. In fact, the apical iron Fe8 of NifB-co was coordinated by C25^E^, while the opposite apical position remained coordinated to NifX, where the structure confirms the interaction of the apical Fe1 with H35^X^ (Fig. 3b), a residue already shown to bind NifB-co by in vitro reconstitution of NifX and NifB-co^27^. Consequently, this structure provides a snapshot of the precise moment when NifB-co is handed over from NifX to the NifEN complex for further maturation (Fig. 3e). We also generated a structural model using Boltz^28,29^ that allowed for the inclusion of NifB-co, yielding a very similar binding as observed experimentally (Extended Data Fig. 6). Interestingly, in this 3D class, the opposite side of NifEN has basically no defined density for NifX except for its C terminus. Here, NifB-co is bound at the same position and in the same orientation as in the pure NifEN classes (Figs. 1c and 2a). As was shown for the reactivity of the structurally similar MoFe protein, the two copies of NifEN in the heterotetramer, thus, do not appear to operate in synchronicity^30,31^.

## Discussion

### NifB-co maturation on NifEN

With the present study, we aimed to understand the final maturation step of the nitrogenase FeMo-cofactor that occurs on the scaffold NifEN. The structural snapshots of cofactor-bound NifEN and the NifENX complex shed light on the initiation phase of this process. After its synthesis on NifB, NifB-co is transferred to NifX, which binds tightly to NifEN but retains dynamics through its flexible C terminus. While the core domain of NifX does not bind rigidly to the NifE subunit, its cargo is first contacted through the flexible N terminus of NifE, ligated through cysteine C25^E^. Concomitant with the delivery of the cluster to NifE, C15^E^ displaces an apical ligand from H35^X^ and the N terminus of NifE folds around the cluster near the protein surface. While, at this stage, we still observe density for the C terminus of NifX, confirming its presence, the globular domain in the apo state regains full flexibility. The intricate interaction of NifX with NifEN underlines the caution that the cell takes with NifB-co at this point, the product of a costly biosynthesis. It also provides an illustrative example of the key role of the dynamic yet specific interactions that so frequently are essential for protein function.

**Fig. 4:**
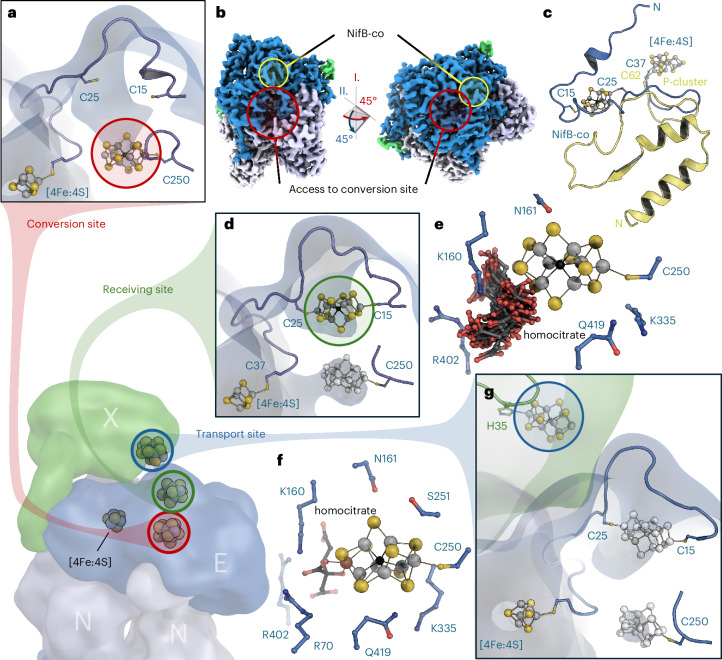
In silico analysis of NifB-co and FeMo-co binding to NifX and NifEN. **a**, Using Boltz-2, a single NifB-co is consistently predicted to bind inside NifE at residue C250^E^, consistent with FeMo-co binding to NifDK (Fig. 2g). **b**, NifB-co-bound NifEN shows two distinct pathways from the protein surface to the internal conversion site, one close to the NifE–NifN interface (left) and another between the three domains of NifE (right). **c**, In the homologous MoFe protein (yellow), both of these pathways are blocked by the extended N terminus of NifD that differs strongly from the NifB-co-binding N terminus of nNifE. **d**, Only if two NifB-co clusters are used with Boltz-2 does the second one bind to the receiving site through cysteine C15^E^ and C20^E^. In this prediction, the N terminus of NifE is not tightly folded onto the cofactor, making it more accessible from the outside. **e**, With inclusion of homocitrate, the ligand clusters strongly around the free apical iron of the NifB-co in the conversion site. **f**, If FeMo-co is used with homocitrate instead of NifB-co, the prediction becomes highly consistent, with homocitrate binding to the terminal Mo ion, surrounded by positively charged residues. **g**, Only when a third NifB-co is included in the Boltz-2 prediction is the transport site at H34^X^ of NifX also occupied, consistent with the experimentally observed binding mode (Fig. 3).

With the transfer of NifB-co to NifEN, however, the work of this maturase complex only begins. NifEN-bound NifB-co is located at more than 12 Å from C250^E^, which in turn sits in a spacious cavity within the protein. Thus, with the advent of novel prediction models, we explored possibilities for the further events that occur on NifEN. Using the most recent MIT Boltz-2 model^29^, we included the intrinsic [4Fe:4S] cluster together with NifEN, as well as NifB-co and the reaction product FeMo-co. Boltz-2 produced a highly accurate model for NifEN with bound [4Fe:4S] cluster, with an r.m.s.d. from the cryo-EM structure for all atoms of 1.2 Å. However, when including NifB-co, we did not observe binding of the cluster to the N terminus of NifE in a single instance of 75 runs. Instead, the tool consistently placed the cofactor precursor inside the protein, into the internal cavity formed by NifE and NifN and, in most cases, directly coordinating residue C250^E^ through an apical iron ion (Fig. 4a). The cavity holding the cluster showed a distinctly positive electrostatic surface potential and access pathways to the binding site were apparent both from the receiving loop with residues C15^E^ and C25^E^ and the surface of NifE (Fig. 4b). This second opening exists in NifE because, while the αIII domain of NifE sealed off the cofactor cavity very similarly to the situation in NifDK, the N terminus of NifD in the enzyme folds very differently from that of NifE, tightly closing access to the cofactor-binding cavity in Mo-nitrogenase (Fig. 4c).

To address the discrepancy in cofactor position between the experimental and predicted structures of NifEN, we then introduced a second NifB-co moiety into the predictions. Indeed, this cluster now consistently bound to residues C15^E^ and C25^E^ with its apical iron Fe1, largely recreating the experimentally observed binding mode (Fig. 4d). This finding raised the question whether the predicted internal binding site for NifB-co was hallucinated because of NifEN similarity to NifDK or whether given the proximity to the essential C250^E^ provided an actual insight into the function of NifEN. Therefore, in further runs, we added homocitrate, as well as the product of the maturation process, FeMo-co having homocitrate attached. Boltz-2 placed homocitrate close to Fe8 at the other apex of NifB-co, although a direct metal coordination was not always observed (Fig. 4e). Similarly, FeMo-co was predicted to bind in the same position, now with a more consistent binding mode for homocitrate that was supported by a tight hydrogen-bonding network to positively charged residues around the trianionic organic ligand (Fig. 4f).

While mapping out two possible binding sites on NifEN, this in silico study so far did not address NifX. Including NifX with the prediction accurately reproduced the binding position and tertiary structure of the cofactor chaperone revealed in the cryo-EM analysis and even reproduced its conformational flexibility relative to NifEN. However, we did not observe binding of NifB-co to NifX rather than to either of the two binding positions on NifEN. Consequently, a third cofactor precursor was introduced in the predictions and only then did we observe binding to NifX, consistently at or near the known ligand H35^X^, in line with our experimental structure (Fig. 4g).

Taken together, the experimental structures of the NifEN(X) complex and the Boltz-2 predicted models outline a defined sequence of events in cofactor handling on NifEN. While the observed sequential binding of NifB-co clusters to the three sites in the complex is not sufficient to conclude on actual affinities, it is notable that the function of the maturase NifEN logically implies a handover of the metal cluster from NifX through the receiving site to the conversion site in NifE. Our experimental structures provide evidence for the first two of these three steps and the consistent prediction of NifB-co binding to the conversion site together with the essential role of residue C250^E^ suggest that the replacement of Fe8 for Mo and the addition of the organic homocitrate moiety occur at this position. It remains to be elucidated how the mature FeMo-co is then extracted by NafY to be shuttled to apo MoFe protein. The increased flexibility of the αIII domain of NifE and its analogy to the open state of this domain in apo NifDK support the hypothesis that this area might act as a lid for cofactor exchange, underlining the close evolutionary relationship between the enzyme and its maturase.

## Methods

### Bacterial strains

*A*. *vinelandii* strains used are listed in Supplementary Table 2 and were constructed using a genetic system previously described in detail^20^. For construction of strain DJ1041 the *A*. *vinelandii* genomic intergenic region between the strong *nifH* promoter and the weaker *nifE* promoter was deleted such that the *nifH* promoter drives the expression of *nifENX*^32^. The *nifE* gene of DJ1041 has a His-tag encoded at its N terminus. Because Fe protein, product of *nifH*, is required for processing NifB-co bound to the NifEN scaffold to produce FeMo-co, DJ1041 produces NifEN containing NifB-co^32,33^. DJ2837 was constructed in the same way as DJ1041 except that the *nifE* region encoding residues 4–25 was deleted and a twin Strep-tag-encoding sequence was placed at the C terminus of *nifN*. NifEN produced by DJ2837 is designated NifE^*^N.

### Cell growth

For protein purifications *A*. *vinelandii* cells were grown at 30 °C in a 150-L custom-built fermenter (W. B. Moore) in a modified Burk medium^34^, containing 1 μM Na_2_MoO_4_ as the Mo source and 5.7 mM ammonium acetate as nitrogen source. Cell growth was followed by simultaneously measuring cell density and ammonium consumption using Nessler’s reagent. Once ammonium was depleted (optical density (OD) of ~2.1), DJ1041 and DJ2837 cells were cultured an additional 2 h before harvesting. For growth curves, 250-ml flasks containing 15 ml of Burk medium, which lacks a fixed nitrogen source, were inoculated with *A*. *vinelandii* cells, previously cultured in the presence of a fixed nitrogen source, to an initial OD_600_ of 0.5 and incubated at 30 °C with continues agitation. Every 2 h, the OD_600_ of each sample was measured using a Tecan 200 microplate reader (Tecan) and corrected to a 1-cm pathlength.

### Protein purifications

NifEN and NifE*N were respectively purified using IMAC or Strep-tag affinity purifications procedures as previously described^35,36^. In brief, *A*. *vinelandii* Strep-tagged NifX was purified from recombinant *Escherichia* *coli* BL21(DE3) transformed with the plasmid pDB2109 following the reported procedure^35^. To generate crude extract from DJ1041, 150 g of cell paste was anaerobically resuspended and lysed in 150 ml of 50 mM Tris buffer pH 8.0 containing 2 mM dithionite, 0.2 mM phenylmethane sulfonyl fluoride (Sigma), 2 μM pepstatin (Sigma) and 2 µg ml^−1^ DNAse (Sigma) and disrupted using a Nano DeBee high-pressure homogenizer under an argon atmosphere. Lysates were centrifuged at 58,000*g* for 50 min at 4 °C and the supernatants anaerobically filtered through a 0.45-µm PES membrane. Clarified crude extract from DJ1041 was passed over a blank 5-ml Strep-Tactin XT column equilibrated in buffer A (50 mM Tris-HCl, 350 mM NaCl and 2 mM Na dithionite, pH 8.0) to remove *A*. *vinelandii* biotin-binding proteins. The flowthrough was then directly applied to a 5-ml Strep-Tactin XT column saturated with Strep-tagged NifX equilibrated in buffer A. The column was then washed with three column volumes of buffer A and the NifENX complex eluted using buffer A containing 50 mM biotin. Biotin was subsequently removed from the NifENX complex sample by passing it over a buffer A equilibrated Cytiva HiPrep 26/10 desalting column. Samples were flash-frozen and stored in liquid nitrogen. For isolation of the NifENX complex, pure NifX was used as bait to capture the NifEN complex produced by DJ1041 using a previously described affinity purification procedure^35^. Apo MoFe protein and Fe protein used for in vitro maturation of apo MoFe protein were prepared as previously described^37^. Quantitation of Fe in protein samples was performed by a Virginia Tech service facility using inductively coupled plasma optical emission spectrometry.

### NifEN-dependent in vitro apo MoFe protein maturation

All reagents were obtained from Sigma-Aldrich. Proteins, solutions and reactions were handled under an argon atmosphere. Reactions were carried out in 9-ml serum bottles. The 1-ml reaction cocktails contained 15 µg of apo MoFe protein, 160 μg of NifEN, 163 µg of NifH, 20 mM dithionite, 20 µM sodium molybdate, 200 µM L-homocitrate, 5 mM ATP, 8 mM MgCl_2_, 30 mM phosphocreatine and 50 µg of creatine phosphokinase in 100 mM Tris buffer pH 8.0. The reactions were initiated with the addition of 15 µg of apo MoFe protein followed by the addition of acetylene (0.1 atm) and incubation for 15 min at 30 °C under continuous shaking. At the end of the incubation time, 300 µl of 0.4 M EDTA was added to quench the reaction The produced ethylene was measured by gas chromatography as described previously^35^.

### EPR analysis

Dithionite-reduced and 5,5′-indigodisulfonic-acid-oxidized EPR NifEN and NifE^*^N spectra were recorded on a Bruker ESP-300 spectrometer with an EMX PremiumX microwave bridge and an EMX^PLUS^ standard resonator in perpendicular mode, equipped with an Oxford Instruments ESR900 continuous-helium-flow cryostat using VC40 flow controller for helium gas. All spectra were normalized to a final protein concentration of ~50 µM. Spectra were recorded using the following conditions: temperature, ~12 K; microwave frequency, ~9.38 GHz; microwave power, 20 mW; modulation frequency, 100 kHz; modulation amplitude, 8.14 G; time constant, 20.48 ms. Each spectrum represents the average of five scans.

### Cryo-EM data collection, processing and model building

The ternary complex of NifENX was diluted to 10 mg ml^−1^, mixed with 0.1% of Fos-choline-10 in an inert gas chamber, transferred into a PCR tube and placed in an anaerobic glass vial before use. Cryo-EM grids were prepared using a Vitrobot Mark IV (Thermo Fisher Scientific). Briefly, 6 µl of protein sample was applied to glow-discharged UltrAuFoil 1.2/1.3 300-mesh grids, blotted for 5 s using filter paper and subsequently plunge-frozen in liquid ethane cooled by liquid nitrogen. Data collection was performed using a 300-kV Krios G4 cryo-transmission EM instrument (Thermo Fisher Scientific) equipped with a Selectris energy filter and a Falcon 4i camera. Images were acquired at a pixel size of 0.586 Å per pixel, with a total dose equivalent to 40 e^−^ per Å^2^ in electron-event representation (EER) format.

The micrographs were processed in cryoSPARC (version 4.7.0)^38^ (Extended Data Fig. 1). The EER data were imported with 40 EER fractions and motion-corrected; the contrast transfer function (CTF) was estimated using patch motion correction and patch CTF estimation. Particle picking was initially performed with a blob picker and extracted using a Fourier downsampling factor of 4, followed by two-dimensional 2D classification. Multiple selections of particle sets were subjected to ab initio reconstruction to generate a high-quality reference volume and an alternative low-quality volume to aid with the identification and exclusion of nonrepresentative particles during heterogeneous refinement. Meanwhile, representative 2D class averages displaying well-defined structural features in various views were selected for template-based picking and the obtained particles were subjected to two rounds of heterogeneous refinement. The particles of the best 3D classes were combined, the duplicates were removed and the particles were reextracted with a Fourier downsampling factor of 2. An additional round of heterogeneous refinement was performed to further remove nonrepresentative particles and the best class was subjected to nonuniform refinement^39^. At this point, there was clear density present alongside NifEN that could be assigned to NifX. Multiple rounds of heterogeneous refinement using 3D classification with a focused mask were able to separate the two species. The final particle sets were subjected to nonuniform refinement with minimization over a per-particle scale, optimized per-particle defocus and per-group CTF parameters switched on, followed by local refinement. The resulting map for NifEN was refined to 2.14 Å with *C*_2_ symmetry; for NifENX, the resolution was 2.16 Å. AlphaFold^40,41^ models for NifE_2_N_2_ and NifE_2_N_2_X were used as starting points for model building, fitted into the density maps using UCSF ChimeraX^42^, built in Coot^43^ and real-space refined in PHENIX^44^. The quality of the structure was validated by MolProbity^45^. Data collection and refinement statistics are summarized in Supplementary Table 1. Figures were generated with PyMOL (Schrödinger) or ChimeraX^42^.

### Reporting summary

Further information on research design is available in the Nature Portfolio Reporting Summary linked to this article.

## Online content

Any methods, additional references, Nature Portfolio reporting summaries, source data, extended data, supplementary information, acknowledgements, peer review information; details of author contributions and competing interests; and statements of data and code availability are available at 10.1038/s41589-026-02179-0.

## Supplementary information

Supplementary Information Supplementary Tables 1 and 2.

Reporting Summary

## Data Availability

Raw data and additional material are available from the corresponding author upon request. The structural models and structure factors were deposited to the Protein Data Bank and the EM Data Bank under accession codes PDB 9IAO, EMD-52782 (NifEN) and PDB 9IAN, EMD-52783 (NifENX), respectively.
