# Supplementary material for: Trafficking of a nitrogenase FeMo-cofactor assembly intermediate
Source: Nat Chem Biol. 2026 Mar 23;22(5):822–8. doi: 10.1038/s41589-026-02179-0 (PMC13128457; doi:10.1038/s41589-026-02179-0)
Supplement: Supplementary file 1 — Supplementary Tables 1 and 2. [file 41589_2026_2179_MOESM1_ESM.pdf]

nature chemical biology

Supplementary information https://doi.org/10.1038/s41589-026-02179-0

Trafficking of a nitrogenase FeMo-cofactor assembly intermediate

In the format provided by the authors and unedited

Supplementary Table 1 | Cryo-EM data collection, refinement, and validation statistics.

**Data Collection**	**$NifE_2N_2$**	**$NifE_2N_2X$**
PDB ID	9IAO	9IAN
EMBD ID	EMDB-52782	EMDB-52783
microscope	Thermo Fisher Titan Krios G4	
detector	Falcon 4i	
magnification	215,000	
voltage (kV)	300	
exposure ($e^{-}/Å^2$)	40	
defocus range (µm)	–0.5 to –2.2	
raw pixel size (Å)	0.572	
number of movies	14,022	
initial particles	6,835,040	
imposed symmetry	C_2	C_1
final particle number	195,878	256,875
resolution (Å)	2.14	2.16
FSC threshold	0.143	0.143
**Refinement**		
Map pixel size (Å)	0.7627	0.7627
Model resolution (Å)	2.19	2.25
FSC threshold	0.5	0.5
Model composition		
Non-hydrogen atoms	15,075	15,755
Protein residues	1,854	1,956
Water	883	789
Ligands	6	6
B factors (Å2)		
Protein	32.57	43.51
Ligand	49.61	106.28
R.m.s. deviations		
bond lengths (Å)	0.006	0.003
bond angles (°)	0.789	0.638
Validation		
MolProbity score	1.32	1.29
clash score	5.84	5.33
poor rotamers (%)	0.00	0.00
Ramachandran plot		
favored (%)	98.64	98.71
allowed (%)	1.36	1.29
disallowed (%)	0.00	0.00

Supplementary Table 2 | *A. vinelandii* strains used in the present work.

DJ0035	*Δ nifE* 132-389
DJ0166	*Δ nifX* 33-77
DJ1041	*pnifH::nifE* (*Δ nifHDKTYΔ nafAB*)
DJ2102	Wild Type
DJ2302	*Δ fdxN* 28-67::Kan^R
DJ2597	*nifE* C250A
DJ2745	*Δ nifE* 4-25
DJ2837	*pnifH::Δ nifE* 4-25 (*Δ nifHDKTYΔ nafAB*)
DJ3166	*Δ fdxN* 28-67::Kan^R*, ΔnifE* 4-25

For strains that carry in-frame deletions the corresponding amino acid residues removed from the encoded protein are numerically indicated after the gene designation. DJ1041 carries a genomic deletion such that the intergenic region between *nifH* and *nifE* (shown in parentheses) is deleted creating a fusion of the *nifH* promoter to the *nifE* gene. The *nifE* gene also encodes a poly-histidine tag at the N-terminus to permit IMAC affinity purification of NifEN. DJ2837 carries the same genomic deletion as DJ1041 and also has a deletion within *nifE* gene removing the codons for NifE residues 4-25. The *nifN* gene produced in DJ2837 encodes a twin Strep-tag at the C-terminus to permit Streptactin-based affinity purification of NifE*N. Strains DJ2302 and DJ3166 carry a deletion within f*dxN* that has been replaced by a non-polar *kan*^{R} cassette. For strain DJ2597, residue C275 has been substituted by A275. Strains DJ0035 and DJ1041 have been previously described [15,32].
